# The Impact of Different Types of Violence on Ebola Virus Transmission During the 2018–2020 Outbreak in the Democratic Republic of the Congo

**DOI:** 10.1093/infdis/jiaa163

**Published:** 2020-04-07

**Authors:** John Daniel Kelly, Sarah Rae Wannier, Cyrus Sinai, Caitlin A Moe, Nicole A Hoff, Seth Blumberg, Bernice Selo, Mathais Mossoko, Gerardo Chowell-Puente, James Holland Jones, Emile Okitolonda-Wemakoy, George W Rutherford, Thomas M Lietman, Jean Jacques Muyembe-Tamfum, Anne W Rimoin, Travis C Porco, Eugene T Richardson

**Affiliations:** 1 Department of Epidemiology and Biostatistics, School of Medicine, University of California San Francisco, San Francisco, California, USA; 2 F. I. Proctor Foundation, University of California San Francisco, San Francisco, California, USA; 3 Institute of Global Health Sciences, University of California, San Francisco, San Francisco, CA, USA; 4 Department of Geography, University of North Carolina, Chapel Hill, North Carolina, USA; 5 Department of Epidemiology, School of Public Health, University of Washington, Seattle, Washington, USA; 6 Firearm Injury Policy and Research Program, Harborview Injury Prevention and Research Center, University of Washington, Seattle, Washington, USA; 7 School of Public Health, University of California Los Angeles, Los Angeles, California, USA; 8 Ministry of Health, Kinshasa, Democratic Republic of Congo; 9 Department of Population Health Sciences, School of Public Health, Georgia State University, Atlanta, Georgia, USA; 10 Department of Earth Systems Science, Stanford University, Stanford, California, USA; 11 School of Public Health, University of Kinshasa, Kinshasa, Democratic Republic of Congo; 12 National Institute of Biomedical Research, Kinshasa, Democratic Republic of Congo; 13 Harvard Medical School, Boston, Massachusetts, USA; 14 Brigham and Women’s Hospital, Boston, Massachusetts, USA

**Keywords:** Ebola virus disease, violence, transmission, Democratic Republic of the Congo, Africa

## Abstract

**Background:**

Our understanding of the different effects of targeted versus nontargeted violence on Ebola virus (EBOV) transmission in Democratic Republic of the Congo (DRC) is limited.

**Methods:**

We used time-series data of case counts to compare individuals in Ebola-affected health zones in DRC, April 2018–August 2019. Exposure was number of violent events per health zone, categorized into Ebola-targeted or Ebola-untargeted, and into civilian-induced, (para)military/political, or protests. Outcome was estimated daily reproduction number (*R*_*t*_) by health zone. We fit linear time-series regression to model the relationship.

**Results:**

Average *R*_*t*_ was 1.06 (95% confidence interval [CI], 1.02–1.11). A mean of 2.92 violent events resulted in cumulative absolute increase in *R*_*t*_ of 0.10 (95% CI, .05–.15). More violent events increased EBOV transmission (*P* = .03). Considering violent events in the 95th percentile over a 21-day interval and its relative impact on *R*_*t*_, Ebola-targeted events corresponded to *R*_*t*_ of 1.52 (95% CI, 1.30–1.74), while civilian-induced events corresponded to *R*_*t*_ of 1.43 (95% CI, 1.21–1.35). Untargeted events corresponded to *R*_*t*_ of 1.18 (95% CI, 1.02–1.35); among these, militia/political or ville morte events increased transmission.

**Conclusions:**

Ebola-targeted violence, primarily driven by civilian-induced events, had the largest impact on EBOV transmission.

## Teaser

A time-series study of the 2018–2020 Ebola virus disease outbreak in the Democratic Republic of the Congo found both Ebola-targeted and untargeted violence increased transmission, though targeted violence, primarily driven by civilian-involved events, had the largest impact.

**(See the Editorial Commentary by Schieffelin, on pages 1941–2.)**

Of the more than 36 recorded Ebola virus disease (EVD) outbreaks globally [1], the current 2018–2020 outbreak in eastern Democratic Republic of the Congo (DRC) is the first to occur in an active conflict zone, where responders have faced both widespread violence and active community resistance targeting their efforts [2]. Although social resistance has been present in prior EVD outbreaks [3–6], violent events have been rare and not considered a significant impediment to Ebola responses. The current DRC outbreak, as World Health Organization (WHO) Director-General Dr Tedros Adhanom Ghebreyesus describes, is different: “We’re not just fighting Ebola virus; we’re fighting insecurity, violence, misinformation, mistrust, and the politicization of an outbreak” [7].

Since Wannier and colleagues first estimated the impact of violent events on Ebola virus (EBOV) transmission in eastern DRC [8], other groups have further characterized the effects of violence through specific event analyses and interviews [9–11]. Beyond this descriptive work, there has yet to be any research that explores the impact of different types of violent events on EBOV transmission, and specifically whether violence that directly targets Ebola response efforts may impact transmission differently than violence not targeted towards the Ebola response.

There are several potential ways in which both targeted and untargeted violence against Ebola response efforts can increase EBOV transmission. Targeted violent events can directly interfere with the overall response effort by injuring health care workers, damaging Ebola treatment facilities, and grounding staff, which affects epidemiological and other response activities, including safe burials and vaccination. EBOV transmission can also increase through untargeted violent events such as the murder of civilians by armed groups, uprisings from political campaigns and elections, and ville morte events, where an entire city is forcibly shut down for several days in protest and, often, commemoration of murdered victims. As a result of violence, EBOV-infected individuals remain in the community and become increasingly viremic, which places community members (the primary caregivers) at greater risk of acquiring EVD [12, 13].

The current outbreak is occurring in the context of entrenched conflict involving multiple armed groups, divisive national elections, and community distrust of the Ebola response effort, phenomena which have their roots in (neo)colonial aggression [14, 15]. Attacks by and between armed groups, political protests, and violence against Ebola containment teams and facilities have occurred frequently and have been disruptive to response efforts, which then contributes to increased EBOV transmission [2, 16–21]. Because events directed against Ebola response efforts have the potential to create large and long-lasting disruptions, we hypothesized that there is the largest increase in EBOV transmission from Ebola-targeted violent events as compared to Ebola-untargeted events. In order to eventually model the distal, historical determinants of EBOV transmission dynamics, we must begin at the end: with an understanding of the differential effects of path dependent violence as a proximal determinant of increased transmission.

## METHODS

### Study Design, Setting, and Participants

The 2018–2020 EVD outbreak in eastern DRC was officially declared on 1 August 2018 (although it most likely started earlier in May 2018), and, as of 5 August 2019, it had affected 26 health zones in the North Kivu, South Kivu, and Ituri provinces of DRC. Our target population included individuals who live in health zones (ie, subprovincial geographic units of health administration, equivalent to what are called health districts in other countries [22]) where there had been at least 1 confirmed or probable EVD case associated with the current outbreak from April 2018 to August 2019. The study base was a dynamic and open cohort, meaning that participants moved freely between the defined geographic areas that were affected by the outbreak. We had access to 3 independent data sources, each of which measured the exposure or outcome variable at the health-zone level during the study period (details below).

### Measurement

The outcome was the daily estimated reproduction number (*R*_*t*_). The reproduction number is the expected number of cases generated by a single case at time point (*t*) after the index case at the start of the epidemic. It is similar to the idea of a basic reproduction number, *R*_*0*_, but loosens the standard requirements that it is the expected number of cases at the outset of an epidemic in a completely susceptible population. For an outbreak to persist, the reproduction number needs to meet or exceed 1.0, the epidemic threshold of 1 additional case per infected person. The DRC Ministry of Health produces daily situation reports of incident EVD cases. These were used to collect data for the following geographic levels: overall outbreak area, province, and health zone. For each case, we collected data on date of case report and diagnosis classification (reported as probable or confirmed). These daily case counts were summed on a weekly basis and confirmed with the weekly WHO situation reports.

We then calculated the daily *R*_*t*_, using the Wallinga-Teunis method. Briefly, this allows for a likelihood-based estimate of *R* using pairs of cases rather than the entire infection network. We assumed a γ-distributed serial interval with a mean of 14.5 days (standard deviation of 5 days) [23]. The EVD case counts by day (*d*) are then used to infer the reproduction number for each case by weighting each potential transmission link by the density of the serial interval. We estimated the initial reproduction number (*R*_*0*_) and subsequently updated date of case report for each EVD case to calculate daily *R*_*t*_ = *R*_*0*_ e^−*td*^. More details can be found elsewhere [24, 25].

The time-varying exposure was the number of violent events occurring in a health zone over the specified time period. The Armed Conflict Location and Event Data Project (ACLED) is a US-based nonprofit organization that aggregates data for all countries in the world related to political violence, protest, conflict, and other violent events that are reported by a wide range of sources (websites such as Kivu Security Tracker, mobile apps such as Twitter, local and international media such as radio or television, and more) [26, 27]. We extracted data from the ACLED database and organized them to include the following variables: event date, event type, subevent type, actor, country, province, health zone, GPS coordinates, fatalities, original reporting source, time stamp, and text-string of event description. A violent event was defined by the ACLED database. We excluded events that were not potentially disruptive to the Ebola response (eg, voluntary repatriation, signed treaties, public speeches). We cross-referenced and classified the violent event GPS coordinates into the health zones where they occurred [28]. In addition, we created a database of violent events described in the WHO situation reports and cross-referenced these data with our ACLED database to ensure the representativeness of the sample [2, 26]. We collected these data from 2 independent data sources in order to obtain a comprehensive and representative sample of violent events.

Data were publicly available and deidentified, so ethics committee approval was not needed.

### Statistical Analysis

We calculated the daily *R*_*t*_ as a continuous variable for the overall outbreak and by health zone. We also calculated the daily number of violent events as an ordinal variable that occurred in the overall outbreak area and by health zone; these geographic units were the first of 3 hierarchical exposure levels used in our analyses. For the second exposure level, we categorized the events into either Ebola-targeted or Ebola-untargeted, creating 2 exposure variables. These categories were created by searching through the violent event descriptions for key words and phrases indicating a direct relationship to the Ebola response (words such as Ebola, treatment center, EVD, Ebola virus [EBOV], Ebola Treatment Centers [ETC], Centres de Traitement d’Ebola [CTE], health care worker [HCW], etc. as well as common alternate or misspellings of these terms). Given that every violent event was also described by a subevent type, we further categorized each event within the Ebola-targeted and Ebola-untargeted categories into subcategories, which formed 3 exposure variables (and our third exposure level): (1) civilian involvement, (2) (para)military or political, and (3) protests. These were defined as follows: Ebola-targeted were those targeting the Ebola response efforts (eg, violence targeting treatment centers, Ebola funerals, relief workers, or Ebola victims); Ebola-untargeted events were not directly related to Ebola response efforts. The subdivisions were defined as follows: (1) militia or political were those involving the military, armed groups, or political conflict (eg, battles or conflicts between armed groups, seizure of weapons, retaking or forceful occupation of land, conflicts between long-standing ethnic or political violence); (2) civilian involvement were those involving civilians (occupation of villages, civilian-led violence against Ebola efforts, local rapes and murders, forced labor, militia violence targeting civilians motivated to cause fear); and (3) protests (ville morte protests and Ebola-related protests by health care workers were grouped separately). The coding for categorization of each exposure was independently reviewed by 2 coauthors (J.D.K. and S.R.W.). For each exposure level and associated exposure variables, we calculated the daily number of violent events that occurred by health zone. We generated our time-varying exposure to violence variable by counting the number of daily events reported in an interval of 7, 14, 21, 28, or 35 days prior to disease onset.

We fit linear time-series regression using multiple linear regression models to assess the relationship between the time-varying violence variable and outcome (*R*_*t*_). To adjust for autocorrelation in the time series, we performed a block time-series bootstrap (block size = 7 days) to adjust the standard errors of the estimates for each specified time period. We fit an unadjusted model for each hierarchical exposure category (overall, 2 Ebola-targeted categorizations, 3 further subtype categorizations) and for each of the 5 intervals of violence exposure. We then performed adjusted analyses estimating the impact of each categorization, while including as a covariate all other reported events at the same hierarchical level. For example, to estimate the adjusted effect of the Ebola-targeted category, we fit a model with both Ebola-targeted and Ebola-untargeted as covariates. We repeated this procedure at the subcategory exposure level. We then ran a Bartels trend test to assess the linearity of the relationship between number of events and EBOV transmission. In addition, we ran sensitivity analyses with a range of transmission-mixing parameters (ω = 0.0–1.0, where 0.0 indicated no mixing between health zones and 1.0 indicated perfect mixing across all health zones) (Supplementary Figure 1) but used a mixing parameter of ω = 0.3 as the best fit parameter based on transmission dynamics and in consultation with local experts. We also ran a sensitivity analysis that excluded WHO-cited violent events that did not overlap with the ACLED dataset (Supplementary Figure 2). We considered a *P* value of < .05 as statistically significant. Analyses were conducted in the statistical software *R* 3.5.3 (R Foundation for Statistical Computing).

## RESULTS

**Figure 1.**
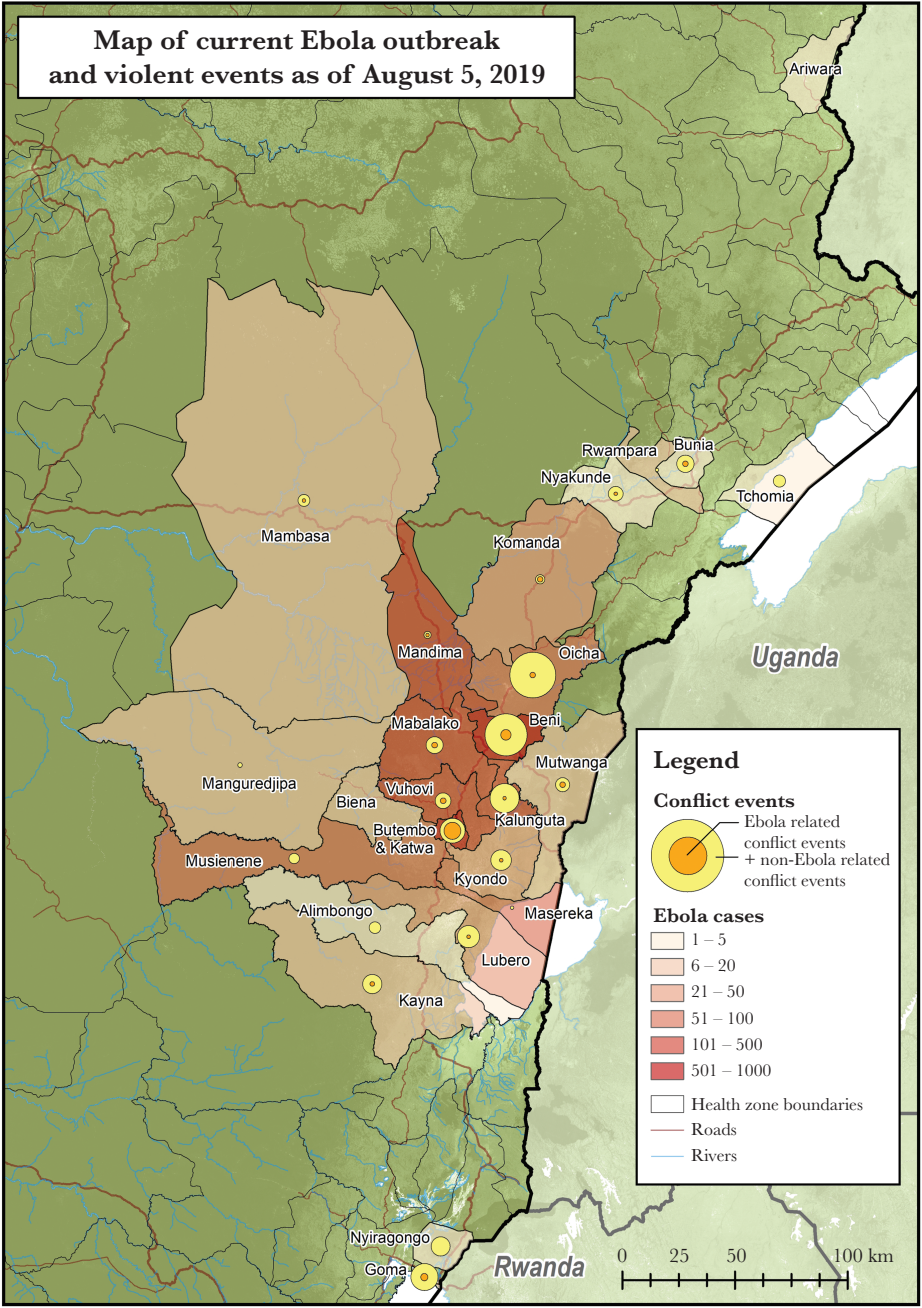
Map of Ebola virus disease cases and potentially disruptive events by health zone.

**Figure 2.**
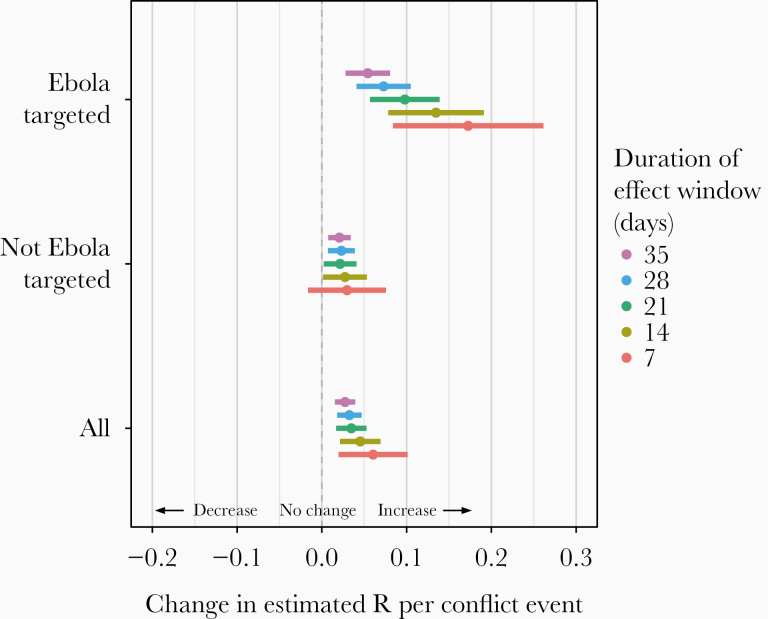
Associations per event of potentially disruptive events with the daily estimated reproduction (*R*_*t*_) number for the outbreak and based on being targeted or not targeted towards the Ebola response.

As of 5 August 2019, there were 2774 probable and confirmed EVD cases reported across 3 provinces and 26 health zones in northeastern DRC [2]. The daily *R*_*t*_ by health zone is shown in Supplementary Figure 3. Over this time period, there were 656 violent events. Sixty-two were Ebola-targeted while 594 were not targeted towards Ebola. Violent events were generally concentrated in the most eastern part of the outbreak-affected health zones, which tended to coincide with zones reporting high concentrations of EVD cases (Figure 1). Each case was affected by a mean of 2.92 violent events in the past 21 days (range, 0–16; interquartile range, 1–5). The average *R*_*t*_ (Ebola virus transmission rate) was 1.06 (95% confidence interval [CI], 1.02–1.11). The effect of each violent event over 21 days on *R*_*t*_ was to increase it by 0.04 (95% CI, .02–.05). This effect was larger among Ebola-targeted events and similar among Ebola-untargeted events (Figure 2).

Among the Ebola-targeted events, 42 involved civilians (eg, attacks on health workers or funerals), 9 were military or politically related (eg, actions taken by militias against ETCs to terrorize communities), and 11 included peaceful protests (eg, health care workers striking for hazard pay, communities demanding a stronger Ebola response). Ebola-targeted events corresponded to a relative increase in *R*_*t*_ of 1.52 (95% CI, 1.30–1.74) at 21 days. Considering subcategorized violent events, targeted events by civilians towards the Ebola response corresponded to a relative increase in *R*_*t*_ of 1.43 (95% CI, 1.21–1.35). Other subcategorized violent events were not statistically significant.

Among the events not targeted towards the Ebola response, 358 were by civilians (eg, looting, mass displacement, forced labor, rape, murdered civilians), 218 were by the military or related to politics (eg, battles between armed groups), and 18 were due to protests, including ville morte events (eg, a general blockade of town activity). While there were more reported events not directly targeting Ebola response efforts, they had a smaller impact, corresponding to a relative increase in *R*_*t*_ of 1.18 (95% CI, 1.02–1.35). Untargeted events connected to the milita/politics or ville morte (protests) had a smaller impact. The relative increase in *R*_*t*_ was 1.18 when there were at least 4 milita or politics events (95% CI, .98–1.37). Ville morte events were also associated with an increased *R*_*t*_ (1.13 for at least 2 events; 95% CI, .96–1.15).

**Figure 3.**
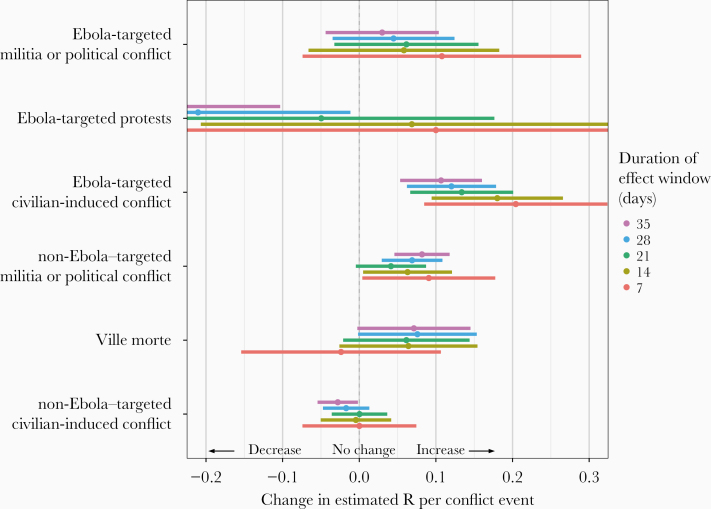
Associations per event of potentially disruptive events with the daily estimated reproduction (*R*_*t*_) number for the subcategorizations targeted and not targeted towards the Ebola response.

These findings were consistent when aggregated by subcategory, regardless of the illustrated percentile (Figure 3). It should be noted that the time period used to capture the effect of violent events impacted the findings for some categories. As demonstrated in Figure 3, shorter periods generally had wider confidence intervals, some of which crossed zero. We chose to focus on the 21-day period because it was longer than a serial interval and was therefore better suited to capture transmission changes.

A mean of 2.92 violent events resulted in a cumulative absolute increase in *R*_*t*_ of 0.10 (95% CI, .06–.15). More violent events were associated with increased EBOV transmission (trend test, *P* = .03). These cumulative effects of a growing number of violent events can be more dramatic and rather meaningful. Over one 21-day interval, for example, 16 events occurred and corresponded with a relative increase in *R*_*t*_ of 1.60 (95% CI, 1.34–1.85). We considered the number of violent events over all 21-day intervals by a range of percentiles and evaluated its relative impact on *R*_*t*_. We illustrate the number of events and relative increases in *R*_*t*_ occurring in the 95th percentile of reported events (see Table 1 for full details).

**Table 1.** The Number of Events and Associated Relative Increases in *R*_*t*_ in Each Percentile of Reported Events

Percentile	Ebola-Targeted	Ebola-Untargeted	EBOV Civilian Induced	Non-EBOV Militia/Political	Non-EBOV Ville Morte
50th percentile					
No. events in the past 21 days	0	1	0	1	0
Relative change in *R* (95% CI)	Ref	1.02 (1.00–1.04)	Ref	1.04 (1.00–1.09)	Ref
75th percentile					
No. events in the past 21 days	1	3	1	2	0
Relative change in *R* (95% CI)	1.10 (1.06–1.15)	1.07 (1.01–1.13)	1.14 (1.07–1.21)	1.09 (.99–1.19)	Ref
90th percentile					
No. events in the past 21 days	3	6	2	3	1
Relative change in *R* (95% CI)	1.31 (1.18–1.44)	1.14 (1.01–1.26)	1.28 (1.14–1.43)	1.13 (.99–1.28)	1.07 (.98–1.15)
95th percentile					
No. events in the past 21 days	5	8	3	4	2
Relative change in *R* (95% CI)	1.52 (1.30–1.74)	1.18 (1.02–1.35)	1.43 (1.21–1.64)	1.18 (.98–1.37)	1.13 (.96–1.31)
98th percentile					
No. events in the past 21 days	6	10	3	5	4
Relative change in *R* (95% CI)	1.62 (1.36–1.89)	1.23 (1.02–1.44)	1.43 (1.21–1.64)	1.22 (.98–1.46)	1.26 (.91–1.61)

Effects for the Ebola-targeted and Ebola-untargeted events are given, as well as statistically significant subgroupings.

Abbreviations: CI, confidence interval; EBOV, Ebola virus; *R*, reproduction number; Ref, reference.

## Discussion

In this analysis, we have attempted to more precisely identify the different effects of Ebola-targeted versus Ebola-untargeted violence on subsequent EBOV transmission in the current outbreak in the eastern DRC. Ebola-targeted violence had the largest impact on EBOV transmission, and this effect was primarily driven by civilian-induced violence. Untargeted violence also had an impact, though it was smaller than targeted violence; the greatest effect of untargeted violence on transmission was due to (para)military, political demonstrations, and ville morte events. The cumulative effect of violent events can have devastating consequences on transmission, as we observed when 16 events over a 21-day interval increased EBOV transmission by 60%.

There are several mechanisms through which violence may exacerbate EBOV transmission, including the interruption of contact tracing, reduction of the uptake as well as timeliness of vaccination, decrease in care-seeking behavior or late presentation to treatment centers, impeded movement in and out of areas, and the violence against Ebola responders that can lead to their evacuation (and thus disruption of the vital care they provide)—all of which were observed by the senior author (E. T. R.), a physician-anthropologist who worked as an Ebola case management consultant for the WHO in North Kivu. However, these impacts may differ based on the type of violent event. Violence that directly targets Ebola treatment centers (or ETCs), such as the burning of ETCs, likely have an outsized negative impact on Ebola treatment and vaccination and possibly decrease care-seeking behavior as people may become fearful of being transferred to centers that are targeted for violence. Other violent events such as the disruption of funerals and other culturally sensitive activities of Ebola-infected people represented challenges with community acceptance of response efforts, reducing the effectiveness of interventions. Political, (para)military, and other untargeted violence may act primarily through mechanisms associated with instability, for instance by causing displacement of communities, interrupting vaccination efforts, and reducing the ability of aid workers to effectively identify, isolate, and trace cases and to vaccinate contacts. In addition, attacks on civilians by armed groups reduces trust in the United Nations and DRC forces mobilized to protect them, mistrust which extends to Ebola containment efforts [29]. Ville morte and other untargeted events have been documented to completely interrupt vaccination and contact tracing efforts for several days, which may impact the timeliness and efficacy of such control efforts [30, 31]. Mediation analyses are needed to identify which of these mechanisms of violence impact EBOV transmission.

There are several limitations of this analysis. First, we rely upon EVD case report data, which almost certainly has incomplete ascertainment [30, 31]. We assume that cases are missing in an unbiased way across time, but, if they are missing in a nonrandom way across health zones or time, this may have biased our results. However, it is likely that cases are more often unreported in regions with the greatest instability and unrest, which would likely have the effect of biasing our results toward the null if the underreporting in these areas was high enough. In general, EVD outbreaks have a high level of regional heterogeneity and stochasticity, which make attempts to analyze them difficult. Though we have made efforts to conceptually define and categorize violent and potentially disruptive events, conflict settings are inherently chaotic and complex. The nature, causes, and motivations of these events, as well as their magnitude and breadth of impact, may vary greatly. We were able to analyze cases, and thus conflict, at only the health zone level, and so there is a possibility that violent events could be somewhat spatially removed from the active areas of EBOV transmission. Our measures of violent events are dependent upon the events being reported, and it is very likely that many violent events occur in these areas that go unreported. To limit missing events, we compared events reported in the WHO situation reports against events reported in the ACLED database. The WHO situation reports, however, did not offer a comprehensive list of violent events, so we were only able to spot-check the ACLED database. Unmeasured common causes and determinants of violence may also be impacting transmission in ways not considered in this analysis.

Meaningful and effective interventions to increase acceptance of EVD treatment and control efforts must consider the sociopolitical determinants of conflict in the region [32–35]. Indeed, many of the violent events in eastern DRC have their roots in more distal yet critically important histories, including colonialism [36], purposeful underdevelopment [37–39], Cold War client support [35], structural adjustment [40], and illicit financial flows [41]. As such, our future goal is to develop models that incorporate more of the structural features—which have substantial but, as yet, understudied impacts on disease transmission—of the geopolitical and social landscape in which EVD epidemics occur [42].

The current outbreak in eastern DRC is the first time that an EVD outbreak has occurred in an active conflict zone and, thus, creates many difficulties and unique challenges for EVD response and control [43–45]. Previous work has focused largely on problems of community disbelief and mistrust of EVD-response efforts [46]. The types of violence that we identified may have a role in subsequent outbreaks because this is not likely to be the last time that a disease outbreak occurs in a high-conflict setting where there are challenges to providing timely and effective care to afflicted communities.

At the time of this writing, the overall *R*_*t*_ in this current outbreak hovers around 1, indicating that it is near the threshold for fade-out. Thus, this study further underlines how even small changes in transmission can have significant negative impacts upon controlling transmission. Increased violent events in December 2019 and subsequent transmission flareups underscore the ramifications of this study’s findings. Under these circumstances, EVD has the potential to smolder along or become an endemic disease, and until appropriate strategies are developed and implemented to protect health workers, allay conflict and insecurity, protect civilians, and rebuild trust by repairing past injustice, many scholars predict this outbreak may continue for the foreseeable future while Ebola responders struggle to contain it. A broader understanding of historical determinants may be required to stop this outbreak and prevent further ones.

## Supplementary Data

Supplementary materials are available at *The Journal of Infectious Diseases* online. Consisting of data provided by the authors to benefit the reader, the posted materials are not copyedited and are the sole responsibility of the authors, so questions or comments should be addressed to the corresponding author.

jiaa163_suppl_supplementary_figure_1

jiaa163_suppl_supplementary_figure_2

jiaa163_suppl_supplementary_figure_3

## Notes

***Disclaimer***. The funding sources had no role in the writing of the manuscript or the decision to submit for publication.

***Financial support.*** This work was supported by the National Institute of Allergy and Infectious Disease (K08 grant number AI139361 to E. T. R.; and K23 grant number AI135037 to J. D. K.); and the National Institute of General Medical Sciences (R01 grant number GM130900 to T. C. P., J. D. K., and E. T. R.).

***Potential conflicts of interest.***. All authors: No reported conflicts of interest. All authors have submitted the ICMJE Form for Disclosure of Potential Conflicts of Interest. Conflicts that the editors consider relevant to the content of the manuscript have been disclosed.

Presented in part: American Society of Tropical Medicine and Hygiene 68th Annual Meeting, 20–24 November 2019, National Harbor, MD.

## References

[1] Arwady MA, Bawo L, Hunter JC, et al Evolution of Ebola virus disease from exotic infection to global health priority, Liberia, mid-2014. Emerg Infect Dis 2015; 21:578–84. 25811176 10.3201/eid2104.141940 PMC4378496

[2] World Health Organization Regional Office for Africa. Health topics: Ebola virus disease http://www.afro.who.int/health-topics/ebola-virus-disease. Accessed 13 April 2020.

[3] World Health Organization. Factors that contributed to undetected spread of the Ebola virus and impeded rapid containment, 2015 http://www.who.int/entity/csr/disease/ebola/one-year-report/factors/en/index.html. Accessed 13 April 2020.

[4] Cohn S, Kutalek R Historical parallels, Ebola virus disease and cholera: understanding community distrust and social violence with epidemics. PLoS Curr 2016; 8:currents.outbreaks.aa1f2b60e8d43939b43fbd93e1a63a94. 10.1371/currents.outbreaks.aa1f2b60e8d43939b43fbd93e1a63a94 PMC4739438 26865987

[5] Fairhead J Understanding social resistance to the Ebola response in the forest region of the Republic of Guinea: an anthropological perspective. Afr Stud Rev 2016; 59:7–31.

[6] Carrión Martín AI, Derrough T, Honomou P, et al Social and cultural factors behind community resistance during an Ebola outbreak in a village of the Guinean Forest region, February 2015: a field experience. Int Health 2016; 8:227–9. 27059272 10.1093/inthealth/ihw018

[7] Adhanom Ghebreyesus T. Address by Dr Tedros Adhanom Ghebreyesus, director-general. Seventy-second World Health Assembly, Geneva, Switzerland, May 202019 http://apps.who.int/gb/ebwha/pdf_files/WHA72/A72_3-en.pdf. Accessed 13 April 2020.

[8] Wannier SR, Worden L, Hoff NA, et al Estimating the impact of violent events on transmission in Ebola virus disease outbreak, Democratic Republic of the Congo, 2018–2019. Epidemics 2019; 28:100353. 31378584 10.1016/j.epidem.2019.100353 PMC7363034

[9] Wells CR, Pandey A, Ndeffo Mbah ML, et al The exacerbation of Ebola outbreaks by conflict in the Democratic Republic of the Congo. Proc Natl Acad Sci U S A 2019; 116:24366–72. 31636188 10.1073/pnas.1913980116 PMC6883813

[10] Masumbuko Claude K, Underschultz J, Hawkes MT Social resistance drives persistent transmission of Ebola virus disease in Eastern Democratic Republic of Congo: a mixed-methods study. PLoS One 2019; 14:e0223104. 31557243 10.1371/journal.pone.0223104 PMC6762146

[11] Jombart T, Jarvis CI, Mesfin S, et al The cost of insecurity: from flare-up to control of a major Ebola virus disease hotspot during the outbreak in the Democratic Republic of the Congo, 2019. Euro Surveill 2020; 25:1900735. 10.2807/1560-7917.ES.2020.25.2.1900735 PMC6976886 31964460

[12] Towner JS, Rollin PE, Bausch DG, et al. Rapid diagnosis of Ebola hemorrhagic fever by reverse transcription-PCR in an outbreak setting and assessment of patient viral load as a predictor of outcome. J Virol 2004; 78:4330–41. 15047846 10.1128/JVI.78.8.4330-4341.2004 PMC374287

[13] WHO Ebola Response Team. After Ebola in West Africa–unpredictable risks, preventable epidemics. N Engl J Med 2016;375:587–96. 27509108 10.1056/NEJMsr1513109

[14] Richardson ET On the coloniality of global public health. Med Anthropol Theory 2019; 6:101–18. 10.17157/mat.6.4.761 PMC10430880 37588113

[15] Lowes S, Montero E Mistrust in medicine: the legacy of colonial medicine campaigns in Central Africa Discussion paper 12772 London, UK: Centre for Economic Policy Research, 2017.

[16] Dickey C What’s worse than Ebola? Fighting it in a war zone, 14 November 2018 https://www.thedailybeast.com/whats-worse-than-ebola-fighting-it-in-a-war-zone. Accessed 13 April 2020.

[17] Branswell H ‘on a knife edge’: Ebola outbreak threatens to escalate as violence rises. Stat News, 7 May 2019 http://www.webcitation.org/78GuOL4UO. Accessed 13 April 2020.

[18] Branswell H CDC’s Redfield: it could take another year to control Ebola in DRC. Stat News, 15 March 2019 http://www.webcitation.org/78GvnBbNX. Accessed 13 April 2020.

[19] Branswell H Ebola response suffers another setback, as WHO evacuates some staff after attack. Stat News, 17 November 2018 http://www.webcitation.org/78GtZOWVV. Accessed 13 April 2020.

[20] Branswell H Ebola response is working, WHO director-general says, amid criticism and violence. Stat News, 14 March 2019 http://www.webcitation.org/78GwJ4itx. Accessed 13 April 2020.

[21] Richardson ET, Barrie MB, Kelly JD, Dibba Y, Koedoyoma S, Farmer PE Biosocial approaches to the 2013-2016 Ebola pandemic. Health Hum Rights 2016; 18:115–28. 27781004 PMC5070685

[22] United Nations Population Fund (UNFPA). End line evaluation of the H4+ joint program Canada and Sweden (SIDA) 2011–2016: Democratic Republic of the Congo. Country case study. New York: UNFPA, 2017 https://www.unfpa.org/sites/default/files/admin-resource/H4JPCS_DRC_Country_Note_Final.pdf. Accessed 13 April 2020.

[23] Aylward B, Barboza P, Bawo L, et al Ebola virus disease in West Africa--the first 9 months of the epidemic and forward projections. N Engl J Med 2014; 371:1481–95. 25244186 10.1056/NEJMoa1411100 PMC4235004

[24] Kelly JD, Worden L, Wannier SR, et al Projections of Ebola outbreak size and duration with and without vaccine use in Équateur, Democratic Republic of Congo, as of May 27, 2018. PLoS One 2019; 14:e0213190. 30845236 10.1371/journal.pone.0213190 PMC6405095

[25] Worden L, Wannier R, Hoff NA, et al. Projections of epidemic transmission and estimation of vaccination impact during an ongoing Ebola virus disease outbreak in Northeastern Democratic Republic of Congo, as of Feb. 25, 2019. PLoS Negl Trop Dis 2019; 13:e0007512. 31381606 10.1371/journal.pntd.0007512 PMC6695208

[26] Armed Conflict Location and Event Data Project. https://www.acleddata.com/. Accessed 13 April 2020.

[27] Raleigh C, Linke A, Hegre H, Karlsen J Introducing ACLED-armed conflict location and event data. J Peace Res 2010; 47:651–60.

[28] United Nations Office for the Coordination of Humanitarian Affairs. Humanitarian data exchange. DR Congo - health zones, 2019 https://data.humdata.org/dataset/dr-congo-health-0. Accessed 13 April 2020.

[29] Richardson ET, McGinnis T, Frankfurter R Ebola and the narrative of mistrust. BMJ Glob Health 2019; 4:e001932. 10.1136/bmjgh-2019-001932 PMC6936462 31908869

[30] Kelly JD, Barrie MB, Mesman AW, et al Anatomy of a hotspot: chain and seroepidemiology of Ebola virus transmission, Sukudu, Sierra Leone, 2015–16. J Infect Dis 2018; 217:1214–21. 29325149 10.1093/infdis/jiy004 PMC6018898

[31] Richardson ET, Kelly JD, Barrie MB, et al. Minimally symptomatic infection in an Ebola ‘hotspot’: a cross-sectional serosurvey. PLoS Negl Trop Dis 2016; 10:e0005087. 27846221 10.1371/journal.pntd.0005087 PMC5112953

[32] Richardson ET, Fallah MP The genesis of the Ebola virus outbreak in West Africa. Lancet Infect Dis 2019; 19:348–9. 30799253 10.1016/S1473-3099(19)30055-6

[33] Richardson ET, Kelly JD, Sesay O, et al. The symbolic violence of ‘outbreak’: a mixed methods, quasi-experimental impact evaluation of social protection on Ebola survivor wellbeing. Soc Sci Med 2017; 195:77–82. 29156248 10.1016/j.socscimed.2017.11.018 PMC5919219

[34] Richardson ET, Barrie MB, Nutt CT, et al. The Ebola suspect’s dilemma. Lancet Glob Health 2017; 5:e254–6. 28193386 10.1016/S2214-109X(17)30041-4

[35] Nzongola-Ntalaja G. The Congo from Leopold to Kabila: a people’s history. London, UK: Zed Books, 2013.

[36] Césaire A. Discourse on colonialism. New York: Monthly Review Press, 1972.

[37] Rodney W. How Europe underdeveloped Africa. London, UK: Bogle-L’Ouverture, 1972.

[38] Amin S.In: McDonagh F, ed. Neo-colonialism in West Africa. New York: Monthly Review Press, 1973.

[39] Fallah MP, Skrip LA, Gertler S, Yamin D, Galvani AP Quantifying poverty as a driver of Ebola transmission. PLoS Negl Trop Dis 2015; 9:e0004260. 26720278 10.1371/journal.pntd.0004260 PMC4697799

[40] Kim JY, Millen JV, Irwin A, Gershman J, eds. Dying for growth: global inequality and the health of the poor. Monroe, ME: Common Courage Press, 2002.

[41] Hickel J. The divide: global inequality from conquest to free markets. New York: W. W. Norton & Company, 2018.

[42] Arthur RF, Gurley ES, Salje H, Bloomfield LS, Jones JH Contact structure, mobility, environmental impact and behaviour: the importance of social forces to infectious disease dynamics and disease ecology. Philos Trans R Soc Lond B Biol Sci 2017; 372:20160454. 28289265 10.1098/rstb.2016.0454 PMC5352824

[43] Claude KM, Underschultz J, Hawkes MT Ebola virus epidemic in war-torn eastern DR Congo. Lancet 2018; 392:1399–401. 30297137 10.1016/S0140-6736(18)32419-X

[44] Garrett L Welcome to the first war zone Ebola crisis 18 October 2018 https://foreignpolicy.com/2018/10/18/welcome-to-the-first-war-zone-ebola-crisis/. Accessed 13 April 2020.

[45] Contagion Live. Is Ebola on the path to becoming endemic in the DRC? 9 November 2018 https://www.contagionlive.com/news/is-ebola-on-the-path-to-becoming-endemic-in-the-drc. Accessed 13 April 2020.

[46] Vinck P, Pham PN, Bindu KK, Bedford J, Nilles EJ Institutional trust and misinformation in the response to the 2018-19 Ebola outbreak in North Kivu, DR Congo: a population-based survey. Lancet Infect Dis 2019; 19:529–36. 30928435 10.1016/S1473-3099(19)30063-5

