# Supplementary material for: The Impact of Different Types of Violence on Ebola Virus Transmission During the 2018–2020 Outbreak in the Democratic Republic of the Congo
Source: J Infect Dis. 2020 Apr 7;222(12):2021–9. doi: 10.1093/infdis/jiaa163 (PMC7661768; doi:10.1093/infdis/jiaa163)

**Supplementary Figure 1.** Sensitivity analysis looking at a range of transmission-mixing parameters (ω = 0.0-1.0) when the duration of the lagged effect is 21 days. ω = 0.0 corresponds to no mixing between zones, and ω = 1.0 corresponds to perfect mixing. **A.** Associations per event of potentially disruptive events with the daily estimated reproduction number for the outbreak and based on being targeted or not targeted towards the Ebola response. **B.** Associations per event of potentially disruptive events with the daily estimated reproduction number for the sub-categorizations targeted and not targeted towards the Ebola response.

a.
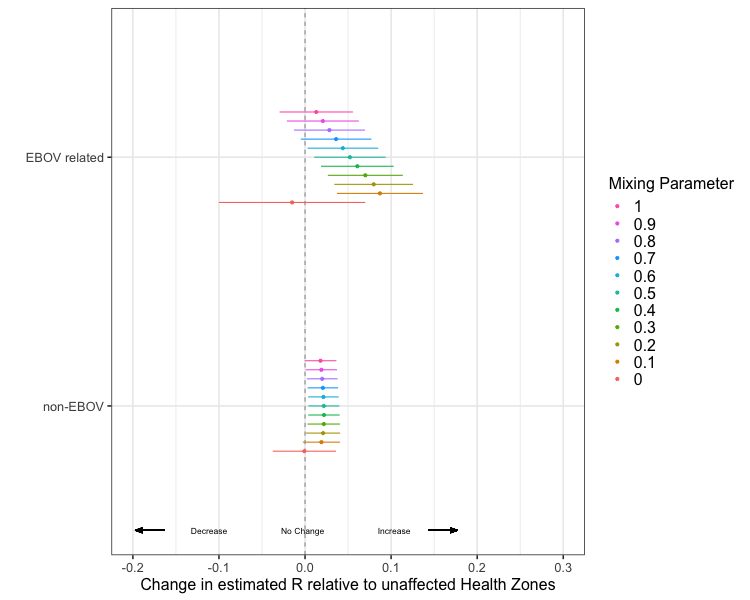
 b.
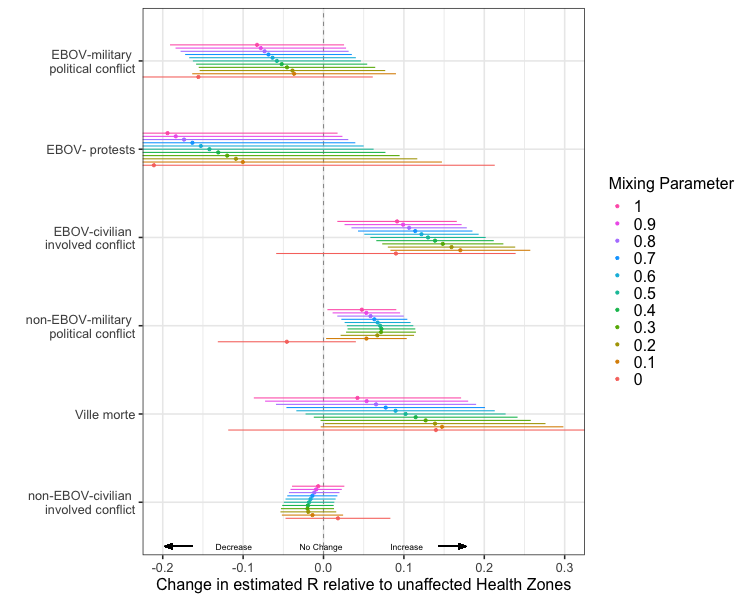

Supplement: jiaa163_suppl_supplementary_figure_1 [file jiaa163_suppl_supplementary_figure_1.docx]
