# Supplementary figures and images for: The Impact of Different Types of Violence on Ebola Virus Transmission During the 2018–2020 Outbreak in the Democratic Republic of the Congo
Source: J Infect Dis. 2020 Apr 7;222(12):2021–9. doi: 10.1093/infdis/jiaa163 (PMC7661768; doi:10.1093/infdis/jiaa163)

**Supplementary Figure 3.** Daily *Rt* by time series from the Wallinga-Teunis Method.


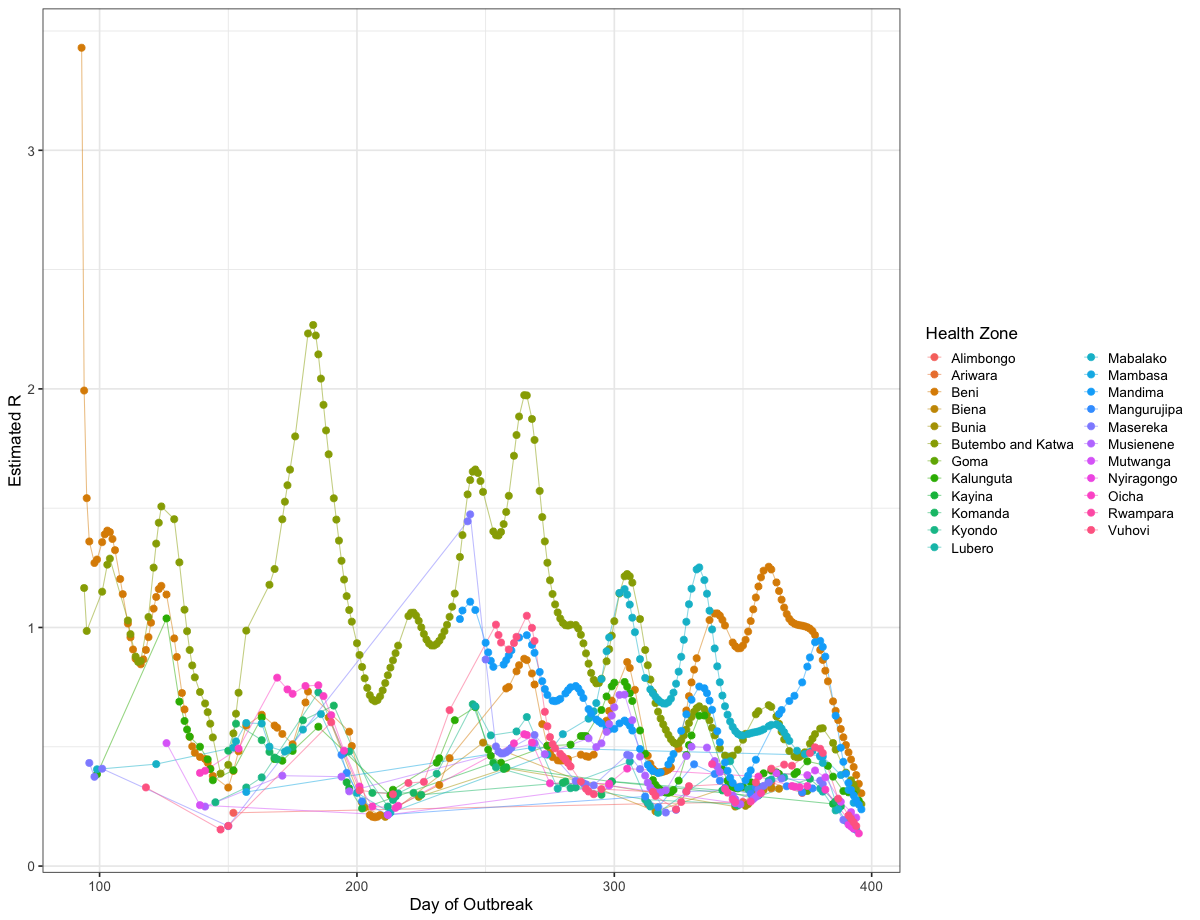

Supplement: jiaa163_suppl_supplementary_figure_3 [file jiaa163_suppl_supplementary_figure_3.docx]
